# Oral health and welfare state regimes: a cross-national analysis of European countries

**DOI:** 10.1111/eos.12049

**Published:** 2013-04-19

**Authors:** Carol C Guarnizo-Herreño, Georgios Tsakos, Aubrey Sheiham, Richard G Watt

**Affiliations:** 1 Department of Epidemiology and Public Health, University College LondonLondon, UK; 2 Departamento de Salud Colectiva, Facultad de Odontología, Universidad Nacional de ColombiaBogotá, Colombia

**Keywords:** adults, Europe, oral health, welfare regimes

## Abstract

Very little is known about the potential relationship between welfare state regimes and oral health. This study assessed the oral health of adults in a range of European countries clustered by welfare regimes according to Ferrera's typology and the complementary Eastern type. We analysed data from Eurobarometer wave 72.3, a cross-sectional survey of 31 European countries carried out in 2009. We evaluated three self-reported oral health outcomes: edentulousness, no functional dentition (<20 natural teeth), and oral impacts on daily living. Age-standardized prevalence rates were estimated for each country and for each welfare state regime. The Scandinavian regime showed lower prevalence rates for all outcomes. For edentulousness and no functional dentition, there were higher prevalence rates in the Eastern regime but no significant differences between Anglo-Saxon, Bismarckian, and Southern regimes. The Southern regime presented a higher prevalence of oral impacts on daily living. Results by country indicated that Sweden had the lowest prevalences for edentulousness and no functional dentition, and Denmark had the lowest prevalence for oral impacts. The results suggest that Scandinavian welfare states, with more redistributive and universal welfare policies, had better population oral health. Future research should provide further insights about the potential mechanisms through which welfare-state regimes would influence oral health.

Political factors have been increasingly recognized as crucial social determinants of health 1–7. Macro-level social policies influence socio-economic inequality, social and cultural capital, and features of the health-care system. They, in turn, have significant effects on psychosocial, material, and behavioural factors related to general and oral health 3,8–13. In broad terms, welfare state institutions and policies affect the distribution of resources that are important to health, such as housing, education, nutrition, and child and health care 14. There is a growing body of research on the association between population health and welfare states 2,7,12,15–17. Studies have consistently shown better performance for health indicators (e.g. infant mortality rates) in Scandinavian (social democratic) welfare states, which have more generous and universal welfare provisions, compared with other welfare regimes 2,5,6.

To analyse some of these issues, the welfare regimes framework has been instrumental as a theoretical perspective. Bambra *et al*. 18 indicated that *welfare state* refers to ‘a particular form of state or specific type of society’ and usually alludes to the role played by the state in social services and benefits, such as education, health, housing, poverty relief, and unemployment, among others. Although the emphasis is placed on the state, other definitions also mention the market and the family. Welfare state is therefore defined as the combination of market, state, and family in providing goods and services within a country 19. According to Esping-Andersen 20, countries can be clustered in *welfare state regimes* based on three principles: decommodification (the extent to which individuals and families can maintain a socially acceptable livelihood regardless of their market performance), social stratification (state commitment to maintain or break down social stratification) and the private–public mix (institutional arrangements for assigning welfare functions to the state, the market, and the family) 13,21–23,20,24,25.

Diverse typologies of welfare state regimes have been proposed according to the type and content of welfare policies 26. The typology most widely used was proposed by Esping-Andersen 20,26–28 and defines three types of welfare states: liberal (most welfare goods and services are provided by the market), conservative (a key role is played by the family, and certain earnings-related welfare benefits are provided by the state), and social-democratic (universal and comprehensive benefits are provided by the state and there is a high level of decommodification). However, some shortcomings of this typology, such as the range of countries and regimes included, and the methodology used 29, have led to the development of alternative typologies. Among these, Ferrera's classification 30 is recognized as one of the most accurate as it considers not only the quantity of welfare provided but how welfare benefits are delivered 31–33. Ferrera identifies four types of welfare states: Scandinavian, Anglo-Saxon, Bismarckian, and Southern 30,32. While the first three types resemble the Esping-Andersen social-democratic, liberal, and conservative groups, respectively, the additional Southern type clusters countries with fragmented welfare benefits. These are: generosity in certain provisions, but weak in others; a marked public–private mix in benefits and services; and some corruption in the selective distribution of cash subsidies 33,30. More recently, the complementary Eastern European welfare state type has been considered, in the social policy literature, to account for specific features of countries that have experienced dramatic changes from a communist welfare state to systems characterized by marketization and decentralization 21. As a result, public health researchers have increasingly used five regime types within Europe: Scandinavian, Anglo-Saxon, Bismarckian, Southern, and Eastern 28,32,34,35.

As stated earlier, populations living in social democratic welfare states that have more generous and universal welfare provisions have better health 2,5,6. However, only one relevant cross-national comparative study has considered how oral health varies by welfare regime. Sanders *et al*. 36 assessed the relationship between welfare states and income inequalities in oral health-related quality of life (OHRQoL) using the Korpi and Palme welfare typology that is based on the generosity and coverage of two social programmes: pensions, and sickness cash benefits. The study compared four countries (Finland, the UK, Germany, and Australia) and showed significantly lower income inequalities in OHRQoL in Germany, where the two social programmes have universal coverage and earnings-related benefits, and larger inequalities in Australia, where benefits are means-tested and the coverage is limited to the low-income part of the population.

To our knowledge, no study has looked at the relationship between welfare regimes and oral health, rather than oral health inequalities; none has used Ferrera's typology to analyse oral health in European countries; and none has included more than one country per welfare state regime. The objective of this study was therefore to assess adults' oral health in a wide range of European countries clustered by welfare state regime, according to Ferrera's typology and the complementary Eastern type.

## Material and methods

### Data source and study sample

We used data from the Eurobarometer 72.3, a cross-sectional survey conducted in 2009 by TNS Opinion & Social at the request of the European Commission. The survey used a multistage, random sampling design to provide representative samples of the adult population in 31 European countries (the 27 Member States of the European Union, three candidate countries – Croatia, Turkey, and the former Yugoslav Republic of Macedonia – and the Turkish Cypriot Community). In each country, all administrative regional units were considered, and sampling points were selected with probability proportional to population size and density. From the sampling points, households were randomly selected, and in each household, one respondent was randomly selected. Data were obtained through face-to-face interviews based on a questionnaire proposed by the European Global Oral Health Indicators Development project.

The survey included separate samples for Great Britain and Northern Ireland, as well as for East and West Germany. We combined the first two as the United Kingdom and the last two as Germany. The total sample of 30,292 individuals, ≥15 yr of age, was used for analyses by country. As the focus was on welfare state regimes, 21 countries classified either in Ferrera's typology or in the Eastern welfare state type were included in the analyses. The total sample for these 21 countries consisted of 21,731 people, with sample sizes in individual countries ranging from 500 to 1,550.

### Oral health outcomes

We considered two self-reported oral health measures: (i) the number of natural teeth, and (ii) the frequency of impacts of oral conditions on daily life. The number of natural teeth was reported through a five-item scale: all; ≥20, but not all; 10–19; 1–9; and no natural teeth. Two binary variables were created: one for not having a functional dentition (fewer than 20 natural teeth) 37, and another for edentulousness (no natural teeth). Only participants ≥45 yr of age were included in the analysis for no functional dentition and edentulousness. In addition, only dentate persons were considered for no functional dentition.

The impacts of oral conditions on quality of life referred to the frequency of the following seven items during the last 12 months: difficulties eating food, difficulties in chewing/biting foods, experiencing pain, feeling tense, feeling embarrassed, avoiding conversation, and reducing participation in social activities. Frequency was measured on an ordinal scale: often, from time to time, rarely, or never. We combined the first two (from time to time and often) and the last two (rarely and never) categories to create a dichotomous indicator for the prevalence of any impact.

### Welfare state regimes

Countries were clustered according to Ferrera's welfare regime typology (Scandinavian, Anglo-Saxon, Bismarckian, and Southern) and the additional Eastern regime. This resulted in five welfare state regimes: Scandinavian (Sweden, Finland, and Denmark), Anglo-Saxon (the UK and Ireland), Bismarckian (Austria, Belgium, France, Germany, Luxemburg, and the Netherlands), Southern (Greece, Italy, Portugal, and Spain), and Eastern (Czech Republic, Estonia, Hungary, Poland, Slovakia, and Slovenia).

### Analysis

We estimated the prevalence rates of oral-health outcomes for each country and welfare state type. The prevalence rates were age-standardized by the direct method, using the whole sample for the 31 countries as a standard population. A poststratification sample weighting (that accounts for nonresponse) and a population-size weighting were used in the analyses to obtain population-based estimates.

## Results

Table1 presents the age-standardized prevalence rates of oral-health outcomes in the different countries. Sweden had the lowest prevalences for no functional dentition (14.4%) and edentulousness (2.94%), and Denmark had the lowest prevalence of oral impacts on daily life (13.8%). On the other hand, Poland had the highest prevalence of edentulousness (26.7%) and Hungary had the highest prevalence for no functional dentition (72.7%). Of the three oral-health outcomes considered, a larger variation in edentulousness existed between countries. There were also variations within groupings of welfare regimes. For example, within the Scandinavian welfare regime, the age-standardized prevalence of edentulousness ranged from 2.94% in Sweden to 12% in Finland, and, in the Eastern regime, from 13.7% in Estonia to 26.7% in Poland.

**Table 1:** Prevalence of oral health outcomes in countries grouped by welfare state regime

Country	*n*	No functional dentition (dentate participants ≥45 yr of age) (%)	Edentulousness (participants ≥45 yr of age) (%)	One or more impacts on daily life, ‘often’ or ‘from time to time’ (%)
Scandinavian (social democratic)				
Sweden	1012	14.40	2.94	17.57
Finland	1017	31.49	12.03	23.05
Denmark	1040	23.26	9.30	13.80
Anglo-Saxon (liberal)				
UK	1354	31.50	13.16	23.41
Ireland	1008	41.21	20.62	19.00
Bismarckian				
Austria	1005	49.16	16.05	29.91
Belgium	1001	43.03	20.63	22.06
France	1000	34.71	10.02	23.79
Germany	1550	38.50	10.65	15.45
Luxemburg	513	37.16	14.23	27.46
the Netherlands	1007	32.15	17.99	16.31
Southern				
Greece	1000	41.67	14.20	25.04
Italy	1032	36.47	10.48	31.60
Portugal	1031	47.82	18.17	28.08
Spain	1003	34.36	11.75	31.09
Eastern				
Czech Republic	1066	47.54	18.01	27.14
Estonia	1011	58.63	13.65	33.36
Hungary	1044	72.69	21.13	26.99
Poland	1000	68.25	26.68	24.62
Slovakia	1006	57.78	21.55	23.80
Slovenia	1031	61.02	17.52	21.82
Not classified				
Cyprus (Republic)	503	32.94	10.99	26.67
Latvia	1018	57.19	10.09	30.69
Lithuania	1026	59.30	11.05	39.99
Malta	500	29.94	13.89	20.69
Bulgaria	1000	55.67	17.19	37.50
Romania	1010	70.11	15.53	46.79
Turkey	1004	45.91	25.16	48.15
Croatia	1000	61.26	13.31	30.16
Cyprus (CY-TCC)	500	32.13	22.77	39.40
Macedonia (FYROM)	1000	60.76	22.47	49.38

The prevalence of oral health outcomes was weighted and age-standardized by the direct method, using the whole sample for the 31 countries as a standard population.

CY-TCC, Turkish Cypriot Community; FYROM; former Yugoslav Republic of Macedonia.

**Figure 1:**
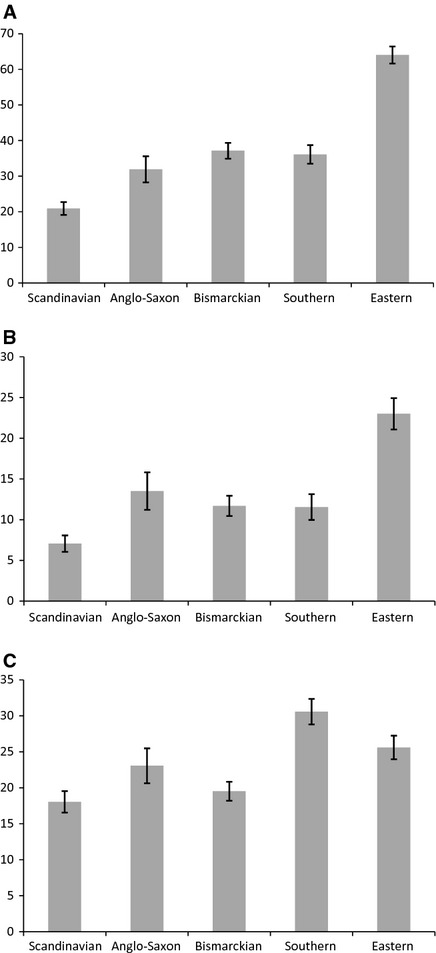
Age-standardized prevalence of oral health outcomes by welfare state regime. (A) No functional dentition (fewer than 20 natural teeth) in dentate participants ≥45 yr of age. (B) Edentulousness in participants ≥45 yr of age. (C) One or more oral impacts on quality of life (all study subjects). Prevalence rates are presented with their 95% CIs.

Figure1 shows the prevalence rates of each oral health outcome by welfare state regime. For the two clinical outcomes (edentulousness and no functional dentition), the Scandinavian regime had a significantly lower prevalence and the Eastern regime had a significantly higher prevalence, when compared with the other welfare regimes. The prevalences of edentulousness and no functional dentition in the other three welfare regimes lay between those of the Scandinavian regime and the Eastern regime, with the Anglo-Saxon regime tending to have a higher prevalence of edentulousness, and a lower prevalence of lack of functional dentition compared with the Bismarckian and Southern regimes. Analyses by sex indicated significant differences between men and women in the prevalence of no functional dentition in the Anglo-Saxon welfare regime and for edentulousness in the Eastern welfare regime. In the Anglo-Saxon countries, 39.6% (95% CI: 33.9–45.3%) of men, but only 24.9% (95% CI: 20.5–29.3%) of women, did not have a functional dentition. Women in the Eastern welfare regime tended to have a higher prevalence (25.1%; 95% CI: 22.6–27.6%) of edentulousness compared with men (20.2%; 95% CI: 17.1–23.4%).

The Scandinavian and Bismarckian welfare regimes showed the lowest prevalence rates for oral impacts on quality of life, whilst the Southern welfare regime exhibited the highest (Fig.1). The Scandinavian regime had a significantly lower prevalence of oral impacts than all other welfare regimes, except for the Bismarckian. Analyses by sex showed that women had a significantly higher prevalence of oral impacts compared with men in the Bismarckian (22.1%; 95% CI: 20.2–24% for women vs. 17%; 95% CI: 15.2–18.9% for men) and also in the Eastern (28.5%; 95% CI: 26.3–30.7% for women vs. 22.3%; 95% CI: 19.8–24.8% for men) welfare regimes. In the other welfare regimes, women also tended to have higher prevalence rates compared with men, although the differences were not significant. Additionally, we compared the prevalence rates of oral impacts using different frequency thresholds. When cases were defined as those that reported ‘often’ in at least one oral impact, the general pattern was similar to that observed in Fig.1C, with the only exception being the Bismarckian regime, which exhibited a slightly higher estimate compared with the Anglo-Saxon regime. In this case, the prevalence rates ranged from 4% in the Scandinavian welfare regime to 6.4% in the Southern welfare regime. The proportion of people reporting ‘from time to time’ in at least one impact ranged from 16.8% in the Scandinavian and Bismarckian regimes to 28.4% in the Southern regime.

## Discussion

Our results support the idea that a welfare regime with more redistributive and universal welfare policies would result in better population oral health. Notably, we found better performance for the Scandinavian welfare regime in all oral health outcomes considered. In addition, complementary analyses, conducted as part of this study using other welfare state typologies (Korpi and Palme 38, Nnavarro & Shi 39, and Bambra 40), found consistent results in terms of the Scandinavian (social democratic) countries, showing significantly lower prevalences for edentulousness and lack of functional dentition (results not shown). In contrast, the analyses reported in this paper showed that the Eastern welfare regime had the highest prevalence rates for these two outcomes, whilst the respective prevalence rates did not vary significantly among the Anglo-Saxon, Bismarckian, and Southern regimes. The prevalence of impacts of oral conditions on daily life was highest in the Southern regime and lowest in the Scandinavian and Bismarckian regimes.

Our findings are in line with previous studies showing that the Scandinavian (social democratic) welfare state regime 2,6,7,12,41,42 has a protective influence on health. Scandinavian countries have lower infant mortality, higher life expectancy, and lower rates of limiting long-standing illness and poor self-rated health 5–7,12,15,41,42. These findings for general health have been attributed to the more generous and universal welfare provisions in the Scandinavian welfare regime and the cumulative effect of its strong redistributive social security system 2,7,15,43. Better population health in Scandinavian countries could also be related to their health policies. They explicitly target the social determinants of health 44 and have a large number of universal health-care services with a high level of decommodification 40. The consistency of our findings with those for general health suggests that the mechanisms linking general health and broad social determinants are also relevant to oral health. In addition, because both edentulousness and lack of functional dentition are cumulative measures of lifetime oral health 45–47, it could be argued that the observed effects of the Scandinavian welfare regime on oral health may operate through diverse mechanisms over the life course.

We found that the Eastern welfare regime had significantly higher prevalences of edentulousness and no functional dentition compared with other regimes. Some previous studies have also found worse population health status in Eastern states 15,28,41. This indicates that the extensive social and political changes experienced by people in the Eastern European countries could have had a negative effect on their oral health, similarly to the observed effect for general health outcomes. Further research with longitudinal data from Eastern countries is needed to confirm this hypothesis. In addition, our findings showed a significantly higher prevalence of edentulousness among women than among men in the Eastern regime, which might be partially explained by the marked intergender gap in life expectancy in Eastern countries 48.

Cultural differences could account for some of the variation in the results between welfare regimes observed in our analyses. For oral impacts on quality of life, cultural factors may partially explain why the Southern regime showed the highest prevalence. It has been argued that higher levels of health complaints found in southern European subjects could be related to cultural issues, such as greater expression of emotions, compared with subjects from other areas of Europe 13,28. A previous study comparing oral impacts on quality of life in people ≥65 yr of age in Britain and Greece indicated cultural effects in the perception of impacts of oral conditions on quality of life 49. The other two oral health outcomes are less likely to differ because of cultural reasons as they are based on the number of natural teeth. However, it is still possible that these measures reveal some cultural differences in the value placed on tooth retention, as reported in previous studies 50,51. Moreover, variation in loss of natural teeth could also be partly attributed to different approaches in dental care practice.

This analysis has strengths worth mentioning. To our knowledge, it is the first study to analyse the effect of different welfare state regimes on oral health status in a wide range of European countries. The macro comparative design, used in this analysis, allows us to assess political determinants that are usually homogeneous within nations 17. Also, by using the same source of information, this analysis has an advantage in terms of precision and comparability as the surveys used the same methodology and time lag for all countries. In addition, the oral health measures considered in our analyses represent different dimensions of oral health. The number of natural teeth can be considered as a measure of lifetime oral health because it captures the cumulative effect of different social determinants of oral health 45–47. On the other hand, the impacts on daily life indicate how clinical oral health affects people physically, psychologically, and socially 52.

The study has limitations that should be considered when interpreting the results. First, the sample sizes are similar for countries with different populations. However, the population size weighting factor used in all analyses corrected for this, as each country is represented in the analyses according to its population size. Second, we do acknowledge that surveys of this nature which are carried out across many different countries are subject to varying measurement error, which may partly influence the results. Third, data for Norway (Scandinavian regime) and Switzerland (Bismarckian regime) were not available, which could have modified some estimates. Nevertheless, it is unlikely that the inclusion of data from these two countries would have considerably altered the general findings. Fourth, the oral health outcomes were self-reported. It has been argued that differences in health perceptions could undermine the validity of cross-national comparisons which are based on self-reported measures 53,54. However, the suitability of subjective health measures for cross-national comparisons has been demonstrated 36,55,56. Moreover, self-reported oral health measures are significantly associated with clinical dental measures and are considered to be valid indicators of oral health 57–60. Finally, the study did not include data on the use or need for prostheses, which would have been a relevant variable considering the outcomes used. Apart from the aforementioned cultural influences, differences in oral impacts on quality of life can partly reflect variations in access and use of dental care services.

It should also be acknowledged that there is no general consensus about an ‘ideal’ welfare regime typology. Based on the existing literature, we included, in the analyses, only countries that have previously been classified in one of the Ferrera's welfare regimes or the additional Eastern type, thereby excluding countries not classified under this typology. We used Ferrera's typology because it accounts for theoretical and methodological weaknesses of previous classifications and it categorizes countries based on different aspects of the welfare provision, and therefore various recent studies have used it to analyse variations in population health and health inequalities 12,27,29,32,34,35,55.

In conclusion, we found significant differences in adults' oral health between welfare state regimes. Our results suggest that characteristics of the Scandinavian countries (particularly Sweden), such as the generosity and universalism of their welfare state benefits, appear to be linked to better oral health outcomes. Also, the Eastern regime showed the highest prevalence rates for edentulousness and no functional dentition, and the Southern regime showed the highest prevalence rate for oral impacts. This is a descriptive study based on macro-level international comparisons and it should be considered as an initial contribution to research on political factors and oral health. By including more social, economic, and political health-care system characteristic variables, future research could gain further understanding on the mechanisms by which welfare-state types influence oral health.

## References

[1] World Health Organization (2008). Closing the gap in a generation: health equity through action on the social determinants of health: final report of the Commission on Social Determinants of Health.

[2] Navarro V, Muntaner C, Borrell C, Benach J, Quiroga A, Rodriguez-Sanz M, Verges N, Pasarin MI (2006). Politics and health outcomes. Lancet.

[3] Borrell C, Espelt A, Rodriguez-Sanz M, Navarro V (2007). Politics and health. J Epidemiol Community Health.

[4] Borrell C, Espelt A, Rodriguez-Sanz M, Burstrom B, Muntaner C, Pasarin MI, Benach J, Marinacci C, Roskam A-J, Schaap M, Regidor E, Costa G, Santana P, Deboosere P, Kunst A, Navarro V (2009). Analyzing differences in the magnitude of socioeconomic inequalities in self-perceived health by countries of different political tradition in Europe. Int J Health Serv.

[5] Navarro V, Borrell C, Benach J, Muntaner C, Quiroga A, Rodriguez-Sanz M, Verges N, Guma J, Pasarin MI (2003). The importance of the political and the social in explaining mortality differentials among the countries of the OECD, 1950–1998. Int J Health Serv.

[6] Chung H, Muntaner C (2006). Political and welfare state determinants of infant and child health indicators: an analysis of wealthy countries. Soc Sci Med.

[7] Chung H, Muntaner C (2007). Welfare state matters: a typological multilevel analysis of wealthy countries. Health Policy.

[8] Bernabe E, Kivimaki M, Tsakos G, Suominen-Taipale AL, Nordblad A, Savolainen J, Uutela A, Sheiham A, Watt RG (2009). The relationship among sense of coherence, socio-economic status, and oral health-related behaviours among Finnish dentate adults. Eur J Oral Sci.

[9] Chaves SCL, Vieira-da-Silva LM (2008). Inequalities in oral health practices and social space: an exploratory qualitative study. Health Policy.

[10] Sisson KL (2007). Theoretical explanations for social inequalities in oral health. Community Dent Oral Epidemiol.

[11] Watt RG (2007). From victim blaming to upstream action: tackling the social determinants of oral health inequalities. Community Dent Oral Epidemiol.

[12] Eikemo TA, Bambra C, Judge K, Ringdal K (2008). Welfare state regimes and differences in self-perceived health in Europe: a multilevel analysis. Soc Sci Med.

[13] Zambon A, Boyce W, Cois E, Currie C, Lemma P, Dalmasso P, Borraccino A, Cavallo F (2006). Do welfare regimes mediate the effect of socioeconomic position on health in adolescence? A Cross-national comparison in Europe, North America, and Israel. Int J Health Serv.

[14] Lundberg O, Yngwe MA, Stjarne MK, Elstad JI, Ferrarini T, Kangas O, Norstrom T, Palme J, Fritzell J, Group NNE (2008). The role of welfare state principles and generosity in social policy programmes for public health: an international comparative study. Lancet.

[15] Karim SA, Eikemo TA, Bambra C (2010). Welfare state regimes and population health: integrating the East Asian welfare states. Health Policy.

[16] Brennenstuhl S, Quesnel-Vallée A, McDonough P (2012). Welfare regimes, population health and health inequalities: a research synthesis. J Epidemiol Community Health.

[17] Muntaner C, Borrell C, Ng E, Chung H, Espelt A, Rodriguez-Sanz M, Benach J, O'Campo P (2011). Politics, welfare regimes, and population health: controversies and evidence. Sociol Health Illn.

[18] Bambra C, Fox D, Scott-Samuel A (2007). A politics of health glossary. J Epidemiol Community Health.

[19] Chung H, Muntaner C (2008). Welfare regime types and global health: an emerging challenge. J Epidemiol Community Health.

[20] Esping-Andersen G (1990). The three worlds of welfare capitalism.

[21] Eikemo TA, Bambra C (2008). The welfare state: a glossary for public health. J Epidemiol Community Health.

[22] Lundberg O (2008). Commentary: politics and public health–some conceptual considerations concerning welfare state characteristics and public health outcomes. Int J Epidemiol.

[23] Myles J, Quadagno J (2002). Political theories of the Welfare State. Soc Serv Rev.

[24] Beckfield J, Krieger N (2009). Epi+demos+cracy: linking political systems and priorities to the magnitude of health inequities-evidence, gaps, and a research agenda. Epidemiol Rev.

[25] Olafsdottir S (2007). Fundamental causes of health disparities: stratification, the welfare state, and health in the United States and Iceland. J Health Soc Behav.

[26] Bambra C (2011). Work, worklessness, and the political economy of health.

[27] Bambra C, Netuveli G, Eikemo TA (2010). Welfare state regime life courses: the development of western European welfare state regimes and age-related patterns of educational inequalities in self-reported health. Int J Health Serv.

[28] Richter M, Rathman K, Gabhainn SN, Zambon A, Boyce W, Hurrelmann K (2012). Welfare state regimes, health and health inequalities in adolescence: a multilevel study in 32 countries. Sociol Health Illn.

[29] Bambra C (2007). Going beyond The three worlds of welfare capitalism: regime theory and public health research. J Epidemiol Community Health.

[30] Ferrera M (1996). The ‘southern model’ of welfare in social Europe. J Eur Soc Policy.

[31] Bambra C (2007). ‘Sifting the wheat from the chaff’: a two-dimensional discriminant analysis of welfare state regime theory. Soc Policy Adm.

[32] Eikemo TA, Huisman M, Bambra C, Kunst AE (2008). Health inequalities according to educational level in different welfare regimes: a comparison of 23 European countries. Sociol Health Illn.

[33] Kim IH, Muntaner C, Vahid Shahidi F, Vives A, Vanroelen C, Benach J (2012). Welfare states, flexible employment, and health: a critical review. Health Policy.

[34] Eikemo TA, Bambra C, Joyce K, Dahl E (2008). Welfare state regimes and income-related health inequalities: a comparison of 23 European countries. Eur J Public Health.

[35] Bambra C, Eikemo TA (2009). Welfare state regimes, unemployment and health: a comparative study of the relationship between unemployment and self-reported health in 23 European countries. J Epidemiol Community Health.

[36] Sanders AE, Slade GD, John MT, Steele JG, Suominen-Taipale AL, Lahti S, Nuttall NM, Allen PF (2009). A cross-national comparison of income gradients in oral health quality of life in four welfare states: application of the Korpi and Palme typology. J Epidemiol Community Health.

[37] Kanno T, Carlsson GE (2006). A review of the shortened dental arch concept focusing on the work by the Kayser/Nijmegen group. J Oral Rehabil.

[38] Korpi W, Palme J (1998). The paradox of redistribution and strategies of equality: welfare state institutions, inequality, and poverty in the Western Countries. Am Sociol Rev.

[39] Navarro V, Shi L (2001). The political context of social inequalities and health. Int J Health Serv.

[40] Bambra C (2005). Cash versus services: ‘Worlds of welfare’ and the decommodification of cash benefits and health care services. J Soc Policy.

[41] Chuang Y-C, Chuang K-Y, Chen Y-R, Shi B-W, Yang T-H (2012). Welfare state regimes, infant mortality and life expectancy: integrating evidence from East Asia. J Epidemiol Community Health.

[42] Bambra C (2006). Health status and the worlds of welfare. Soc Policy & Soc.

[43] Coburn D (2004). Beyond the income inequality hypothesis: class, neo-liberalism, and health inequalities. Soc Sci Med.

[44] Marmot M, Allen J, Bell R, Goldblatt P (2012). Building of the global movement for health equity: from Santiago to Rio and beyond. Lancet.

[45] Aida J, Kondo K, Kondo N, Watt RG, Sheiham A, Tsakos G (2011). Income inequality, social capital and self-rated health and dental status in older Japanese. Soc Sci Med.

[46] Celeste RK, Nadanovsky P, Ponce de Leon A, Fritzell J (2009). The individual and contextual pathways between oral health and income inequality in Brazilian adolescents and adults. Soc Sci Med.

[47] Bernabe E, Marcenes W (2011). Income inequality and tooth loss in the United States. J Dent Res.

[48] Ginter E, Simko V (2013). Women live longer than men. Bratisl Lek Listy.

[49] Tsakos G, Marcenes W, Sheiham A (2001). Cross-cultural differences in oral impacts on daily performance between Greek and British older adults. Community Dent Health.

[50] Davis P (1987). Introduction to the sociology of dentistry: a comparative perspective.

[51] Nassani MZ, Locker D, Elmesallati AA, Devlin H, Mohammadi TM, Hajizamani A, Kay EJ (2009). Dental health state utility values associated with tooth loss in two contrasting cultures. J Oral Rehabil.

[52] Tsakos G, Sheiham A, Iliffe S, Kharicha K, Harari D, Swift CG, Gillman G, Stuck AE (2009). The impact of educational level on oral health-related quality of life in older people in London. Eur J Oral Sci.

[53] Zimmer Z, Natividad J, Lin HS, Chayovan N (2000). A cross-national examination of the determinants of self-assessed health. J Health Soc Behav.

[54] Mitchell R (2005). Commentary: the decline of death–how do we measure and interpret changes in self-reported health across cultures and time?. Int J Epidemiol.

[55] Bambra C, Pope D, Swami V, Stanistreet D, Roskam A, Kunst A, Scott-Samuel A (2009). Gender, health inequalities and welfare state regimes: a cross-national study of 13 European countries. J Epidemiol Community Health.

[56] Robine JM, Jagger C (2003). Creating a coherent set of indicators to monitor health across Europe: the Euro-REVES 2 project. Eur J Public Health.

[57] Borrell LN, Taylor GW, Borgnakke WS, Woolfolk MW, Nyquist LV (2004). Perception of general and oral health in White and African American adults: assessing the effect of neighborhood socioeconomic conditions. Community Dent Oral Epidemiol.

[58] Borrell LN, Baquero MC (2011). Self-rated general and oral health in New York City adults: assessing the effect of individual and neighborhood social factors. Community Dent Oral Epidemiol.

[59] Tsakos G, Demakakos P, Breeze E, Watt RG (2011). Social gradients in oral health in older adults: findings from the English longitudinal survey of aging. Am J Public Health.

[60] Locker D (2009). Self-esteem and socioeconomic disparities in self-perceived oral health. J Public Health Dent.

